# Insights into the Cytoadherence Phenomenon of *Plasmodium vivax*: The Putative Role of Phosphatidylserine

**DOI:** 10.3389/fimmu.2017.01148

**Published:** 2017-09-20

**Authors:** Paulo Renato Totino, Stefanie Costa Lopes

**Affiliations:** ^1^Laboratory of Malaria Research, Instituto Oswaldo Cruz, Fundação Oswaldo Cruz, Rio de Janeiro, Brazil; ^2^Instituto Leônidas e Maria Deane, Fundação Oswaldo Cruz, Manaus, Brazil

**Keywords:** *Plasmodium vivax*, cytoadhesion, rosetting, eryptosis, phosphatidylserine

## Abstract

*Plasmodium vivax* is the most geographically widespread and the dominant human malaria parasite in most countries outside of sub-Saharan Africa and, although it was classically recognized to cause benign infection, severe cases and deaths caused by *P. vivax* have remarkably been reported. In contrast to *Plasmodium falciparum*, which well-known ability to bind to endothelium and placental tissue and form rosettes is related to severity of the disease, it has been a dogma that *P. vivax* is unable to undergo cytoadherent phenomena. However, some studies have demonstrated that red blood cells (RBCs) infected by *P. vivax* can cytoadhere to host cells, while the molecules participating in this host–parasite interaction are still a matter of speculation. In the present overview, we address the evidences currently supporting the adhesive profile of *P. vivax* and, additionally, discuss the putative role of phosphatidylserine—a cell membrane phospholipid with cytoadhesive properties that has been detected on the surface of *Plasmodium*-parasitized RBCs.

## Introduction

*Plasmodium vivax* is the most geographically widespread and the second most prevalent parasite causing malaria in the world, with about 35% of global population living at risk of infection (1) and an estimated 8.5 million symptomatic cases in 2015 (2). *P. vivax* contributes significantly to malaria cases outside of sub-Saharan Africa, where it accounts for 41% of the cases, of which 65% occur in South-East Asia, 19% in Eastern Mediterranean, and the remaining in Western Pacific (9%) and American (7%) regions (2).

Despite its considerable impact in global public health, *P. vivax* was for long time a neglected parasite. The reasons for this scenario include: the low global prevalence [4% (2)], when compared with the most prevalent and lethal malaria parasite, *Plasmodium falciparum*; the failure to adapt to *in vitro* culture conditions; as well as the classically recognized benign profile of infection (3). However, in the last decade, severe cases and deaths due to *P. vivax* infection have remarkably been reported in all endemic regions, driving the attention of the academic community to the real importance of *P. vivax* (4). Moreover, the occurrence of severe forms of malaria in *P. vivax* infections, such as cerebral malaria and placental malaria, which were previously reported to be exclusively associated with *P. falciparum*, suggests that *P. vivax* can, to some extent, present pathogenic profiles similar to *P. falciparum* (5–8).

It is known that the main pathological phenomenon related to high virulence of *P. falciparum* is the sequestration of parasitized red blood cells (pRBC) to vascular endothelium and placenta, which allows late-stage forms of parasite to evade splenic phagocytosis, while provoking host damage by obstructing blood flow and inducing local pro-inflammatory response (9). Additional factors contributing to the pathogenesis of *falciparum* malaria comprise rosetting of pRBC with non-parasitized red blood cells (nRBC) as well as clumping of pRBC mediated by platelets (10). All these cytoadhesive events of *P. falciparum*-pRBC are recognized to be mediated by a large and diverse family of parasite antigens, named *P. falciparum* erythrocyte membrane protein 1 (PfEMP1), that is expressed on the surface of pRBC and shows affinity to several host receptors, including intercellular adhesion molecule 1 (ICAM-1), platelet-endothelial cell adhesion molecule 1, chondroitin sulfate A (CSA), hyaluronic acid (HA), thrombospondin (TSP), and CD36 (10). Since homologous of *var* genes, which encode PfEMP1, have not been identified in other *Plasmodium* species and the cytoadherence of pRBC was not consistently documented in non-*falciparum* malaria, it was believed that the cytoadherence phenomenon of pRBC was restricted to *P. falciparum* infection (11–14). However, some evidences currently support that red blood cells (RBCs) infected by *P. vivax* (Pv-pRBC) also undergo cytoadherence events, as addressed below in the present paper.

## Evidences of *P. vivax* Cytoadhesion

Since Pv-pRBC lack adhesive knob structure and, especially, because all parasite stages can be observed in the peripheral blood of patients, it has been a dogma that *P. vivax* lacks the ability to cytoadhere and, therefore, to sequester. However, in 2010 it was demonstrated, for the first time, that Pv-pRBC are able to cytoadhere *in vitro* to host cells (15). In this study, Pv-pRBC obtained from Brazilian non-severe patient samples were tested by static and flow cytoadhesion assays using human lung endothelial cells (HLEC), monkey brain endothelial cells, and human placental cryosections. Although the number of Pv-pRBC adhered under static conditions was lower than that observed for pRBC infected by *P. falciparum*, the strength of interaction with endothelium was similar. Moreover, it was shown using transfected Chinese Hamster Ovarian (CHO) cells that the binding of Pv-pRBC to ICAM-1-expressing cells was twice as high as to non-transfected cells or CD36-expressing cells and that the adherence to placental cryosections and HLEC was inhibited by soluble CSA, suggesting involvement of both ICAM-1 and CSA in adhesive processes of *P. vivax*. In fact, the adhesive capacity of Pv-pRBC to HLEC and the involvement of ICAM-1 were later recorded in studies using *P. vivax* isolates from Colombia (16). Moreover, a treatment with chondroitinase reversed the adherence of *P. vivax* isolates from the Asia-Pacific region to immobilized CSA, while it was additionally observed that hyaluronidase disturbed Pv-pRBC adhesion mediated by HA (17). Nevertheless, the degree of commitment of each host adhesive receptor studied until now is still a matter of speculation. For instance, in the study with Thai patients described above, all *P. vivax* isolates were adherent to immobilized CSA and HA, but none adhered to ICAM-1 (17), and when *P. vivax* isolates from Brazilian Amazon region was evaluated, a low frequency of pRBC adhesion to ICAM-1 and CSA was observed (18).

Corroborating the adhesive profile of *P. vivax*, it was recently reported that the schizont stage was absent in the peripheral circulation in more than half of Brazilian patients diagnosed with *P. vivax* malaria by blood smears and, even when *P. vivax* schizonts were detected, they were mostly present at low frequency (19). Moreover, *in vitro* maturation of *P. vivax* isolates provided a greater ability of Pv-pRBC to cytoadhere to HLEC than the same isolates before maturation, revealing a higher adhesive capacity of mature forms. These data indicate that *P. vivax* might be sequestered in the deep vasculature and that maturation of late stages of *P. vivax* occur outside peripheral circulation. Actually, more than 50 years ago sequestration of Pv-pRBC was proposed by Field et al. (20), who showed a disappearance of schizonts from the peripheral blood of a *P. vivax* patient. In addition, recent discoveries showing accumulation of *P. vivax* schizonts and gametocytes in the bone marrow (21); detection of a large number of intact Pv-pRBC in the spleen (22); and presence of Pv-pRBC within pulmonary microvasculature from a patient with negative blood smear at the time of death (23) support the hypothesis that *P. vivax* has the ability to sequester.

Although *P. vivax* does not present any protein homologous to PfEMP1, a group of variable proteins (VIR proteins) is expressed by this parasite species (24). In contrast to PfEMP1, VIR proteins are not clonally expressed and can additionally be found within pRBC, indicating initially that these *P. vivax* antigens have different functions from PfEMP1 ones (25). However, based on their variant nature and presence on pRBC surface, the role of VIR antigens in Pv-pRBC adhesion has been evaluated. Thus, computational analysis using a *P. falciparum*-based algorithm revealed putative adhesive protein motifs in VIR proteins (26), which could explain the capacity of *vir* gene (VIR-14) to mediate adhesion of pRBC to ICAM-1 when transfected into a non-adhesive *P. falciparum* line (27). Also, consistent with the participation of VIR proteins in the sequester phenomenon of Pv-pRBC, it was previously demonstrated that antibodies against variants of VIR proteins (VIR-A4 and VIR-E5) partially inhibit adhesion of Pv-pRBC to HLEC (15).

Therefore, there is now a growing body of evidence supporting that *P. vivax* parasites possess adhesive phenotypes. Indeed, besides adhesion to endothelium and placental tissue, it is known that *P. vivax* has the ability to form rosettes, which are defined by the binding of a pRBC with two or more nRBC. Rosetting formation in *P. vivax* infection was described more than 20 years ago (28) and has been shown to be more frequent than in *P. falciparum* infection (29, 30), but few studies have been conducted to investigate this *P. vivax* phenomenon; largely due to the absence of a *P. vivax* continuous culture method. Notwithstanding, it was already demonstrated that rosettes in *P. vivax* infection are formed by interaction of pRBC containing trophozoites, schizonts, or gametocytes with mature RBCs (normocytes), a process that involves glycophorin C receptor present on nRBC surface (30). Furthermore, *P. vivax* rosettes were shown to be stable even under high physiological shear stress and rosette formation was closely associated with induction of an increased rigidity of Pv-pRBC, possibly contributing to sequestration of *P. vivax* in the microvasculature (31). However, differently from *P. falciparum*, both host and parasite antigens intricate on *P. vivax* rosetting as well as the relation of this adhesive phenomenon to the pathogenesis of *vivax* malaria remain unknown.

## The Role of Phosphatidylserine (PS) in Cytoadhesive Phenomena

While some research efforts have been dedicated to identifying *P. vivax* antigens participating in cytoadhesion of pRBC, little attention has been given to host RBC factors that have adhesive potentiality, such as PS. PS is a cell membrane phospholipid usually restricted to the inner leaflet of the lipid bilayer (32), but during apoptotic cell death processes PS is exposed on cell surface, promoting recognition and clearance of dying cells by phagocytes (33). Externalization of PS also occurs in activated platelet and transiently in activated lymphocytes and mast cells, where it is associated with procoagulant activity, homing to sites of inflammation and cell degranulation, respectively (34–36). Furthermore, it has been shown that the presence of PS on external leaflet of cell membrane is a hallmark of suicidal erythrocyte death, named eryptosis (37).

Eryptosis occurs in senescent RBC and can precociously be triggered by a variety of endogenous and xenobiotics stimuli (38, 39). Similarly to apoptosis of nucleated cells, eryptotic processes are characterized by many morphological and biochemical changes, i.e., Ca^2+^ influx, cysteine protease activity, PS exposure, cell shrinkage, and plasma membrane microvesiculation, with externalized PS rendering RBC susceptible to clearance by splenic phagocytes (40). Accordingly, overinduction of PS-exposing eryptotic RBC is believed to contribute to the development of anemia related to several clinical disorders, as reported in both experimental and human malaria (41, 42). But, additionally, PS on RBC surface is also considered one of the factors responsible for thrombo-occlusive events in pathologies such as sickle cell disease, chronic renal failure, retinal vein occlusion, and diabetes; in part, by mediating RBC adherence to endothelium as well as cell aggregation (43–46).

Indeed, adherence of PS-exposing RBC to endothelium is observed *in vitro* under dynamic flow conditions mimicking venular wall shear stress (47, 48) and takes place through interaction of PS with the scavenger receptors CD36 or CXC chemokine ligand 16 (CXCL16) expressed on endothelial cell membrane, as well as with TSP, which is found in the basement membrane and extracellular matrix of endothelium and that can be exposed by vascular injury (45, 48, 49). Moreover, soluble plasma TSP can interact with CD36 and, in this manner, could operate as a bridge to adherence of PS-exposing RBC (50, 51). Such interactions involving PS, CD36, TSP, and CXCL16 have also been involved in RBC-platelet aggregation, agreeing with the presence of CD36 and CXCL16 in platelet membrane (52–54). Thus, if parasite antigens able to provide pRBC adhesiveness are absent, *P. vivax* could explore host adhesive molecules to mediate cytoadhesive events of pRBC.

Interestingly, it has previously been demonstrated that intraerythrocytic plasmodia development progressively induces PS exteriorization on pRBC, with larger exposure at the late stages of parasite maturation (55, 56), which possibly result from eryptosis stimulation. Schizogonic process is described to activate non-selective cation channels in host pRBC membrane, allowing the entry of Ca^2+^ necessary for parasite intracellular growth, which, in turn, leads to the activation of phospholipid scramblases responsible for PS exposure (57). Although PS externalization has not been evaluated in *P. vivax* infection, it was already detected by flow cytometry in RBC infected by *P. falciparum, P. berghei*, and *P. yoelii* (41, 58, 59) and in *P. falciparum*, the binding of late-stage pRBC exposing PS to CD36-expressing cells as well as immobilized CD36 and TSP was inhibited by annexin V, PS-containing liposomes or glycerophosphorylserine—a soluble form of PS (60), indicating that PS could, at least in part, support cytoadhesive phenomena of pRBC in malaria. Consistent with this possibility, a relationship between cytoadhesive activity and PS exposure was also reported when knobby and knobless *P. falciparum* strains, which differentially induce PS externalization on late-stage pRBC, were studied (60) and, more recently, it was also shown that PS-expressing RBC can operate as nuclei for RBC aggregation induced by *P. falciparum*-conditioned medium (61).

Importantly, studies conducted on *P. berghei* ANKA experimental infection with CD36-deficient rodents have demonstrated that CD36 is an essential receptor for sequestration of schizont-stage pRBC, which occurred mostly in the capillaries of lungs and adipose tissue, but not in the brain, where endothelial expression of CD36 is low or absent (62). Indeed, besides being incriminated in acute tissue injury induced by *P. berghei* ANKA-pRBC accumulation in lung (63), CD36 is known as an important receptor mediating pRBC sequestration, non-related to brain and placental tissue in *P. falciparum* malaria (64), and its expression on surface of platelets and RBC has been implicated in clumping and rosetting processes of *P. falciparum*-pRBC, respectively (65, 66). However, it is noteworthy that, in contrast to *P. falciparum*, which expresses the adhesin PfEMP1, but similarly to *Plasmodium chabaudi*, whose late-stage forms undergo CD36-dependent cytoadhesion *in vitro* (67), no putative parasite ligand for CD36 has been identified in genome of *P. berghei*, or even other species of *Plasmodium* displaying cytoadherence phenotypes, such as *P. vivax* (14, 68), reinforcing the premise that alternative pathways, not based on the expression of parasite adhesins, could mediate CD36-dependent cytoadhesion of late-stage pRBC.

**Figure 1:**
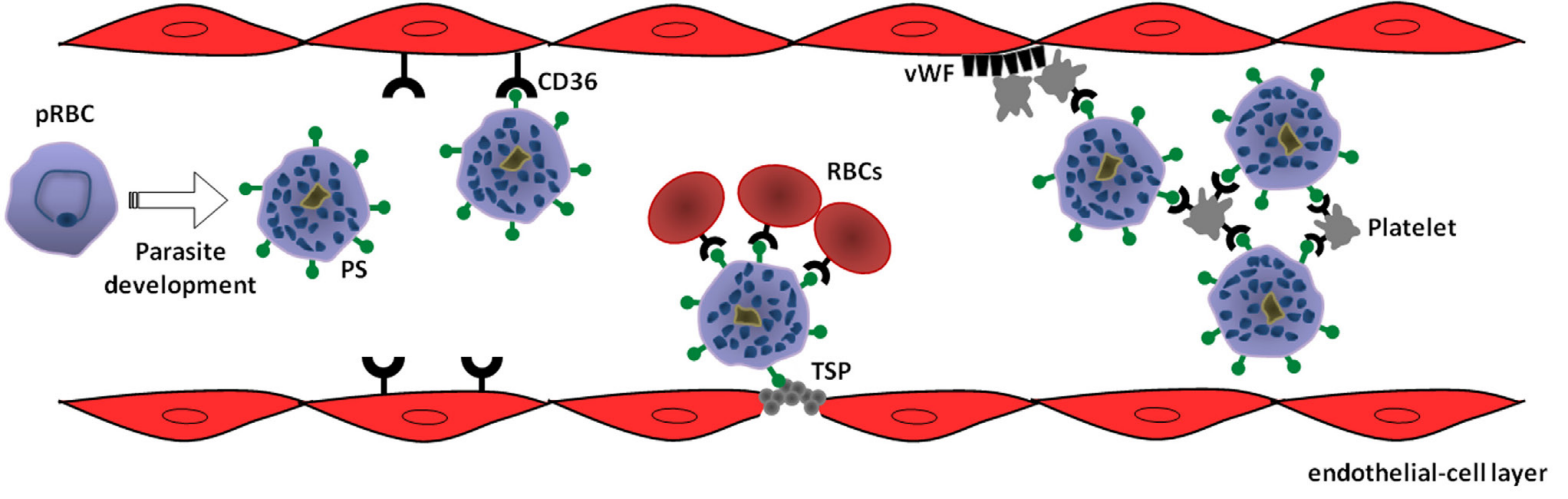
Model of phosphatidylserine (PS) role in cytoadhesive phenomena of *Plasmodium vivax*-parasitized red blood cells (pRBC). Intraerythrocytic parasite development leads to exposure of PS on pRBC surface as a result of suicidal erythrocyte death (eryptosis) induction. In turn, PS mediates sequestration of pRBC to microvasculature through interaction with receptors expressed on endothelial cells, such as CD36, or thrombospondin (TSP) exposed in injured endothelium. In a CD36-dependent manner, PS-exposing pRBC can promote rosetting of non-parasitized red blood cells (RBCs) as well as autoagglutination (clumping) by binding to platelets. Additionally, von Willebrand factor (vWF) can indirectly contribute to the sequestration of PS-exposing pRBC by mediating platelet adhesion at sites of vascular damage (69).

Additional evidences for this proposition are also documented in *P. vivax* malaria. First, a reduction in Pv-pRBC adhesion to HLEC was achieved in the presence of anti-CD36 antibodies, although the small number of samples limited the statistical analysis concerning extension of CD36 participation in *P. vivax* cytoadhesion (15). Second, while studying cellular trafficking and the adhesive propriety of *P. vivax* VIR proteins in *P. falciparum* transgenic lines, it was shown that only one variant of VIR proteins (VIR-14) was exposed at the surface of pRBC, mediating cytoadherence to CHO cells through ICAM-1, but not CD36 (27). Thus, it is tempting to speculate that if antigens encoded by *vir* genes participate in Pv-pRBC adhesive events, it seems that it does not take place through a CD36-dependent mechanism, in which PS could play a role (Figure 1). In view of this possibility, studies evaluating the occurrence of PS externalization in pRBC from *P. vivax* isolates as well as the effect of blocking PS-CD36/TSP interaction on adhesive phenomena of Pv-pRBC may help to confirm the involvement of PS in *vivax* malaria.

## Author Contributions

PT and SL wrote the paper. Both authors read and approved the final version of the manuscript.

## Conflict of Interest Statement

The authors declare that the research was conducted in the absence of any commercial or financial relationships that could be construed as a potential conflict of interest. The reviewer, PG, and handling editor declared their shared affiliation.

## References

[1] World Health Organization. Control and Elimination of Plasmodium vivax Malaria: A Technical Brief. Geneva: World Health Organization (2015).

[2] World Health Organization. World Malaria Report 2016. Geneva: World Health Organization (2016).

[3] Baird JK. Neglect of *Plasmodium vivax* malaria. Trends Parasitol (2007) 23:533–9. 10.1016/j.pt.2007.08.011 17933585

[4] Rahimi BA Thakkinstian A White NJ Sirivichayakul C Dondorp AM Chokejindachai W. Severe *vivax* malaria: a systematic review and meta-analysis of clinical studies since 1900. Malar J (2014) 13:481. 10.1186/1475-2875-13-481 25486908 PMC4364574

[5] Tjitra E Anstey NM Sugiarto P Warikar N Kenangalem E Karyana M Multidrug-resistant *Plasmodium vivax* associated with severe and fatal malaria: a prospective study in Papua, Indonesia. PLoS Med (2008) 5:e128. 10.1371/journal.pmed.0050128 18563962 PMC2429950

[6] Naing C Whittaker MA Nyunt Wai V Mak JW. Is *Plasmodium vivax* malaria a severe malaria? A systematic review and meta-analysis. PLoS Negl Trop Dis (2014) 8:e3071. 10.1371/journal.pntd.0003071 25121491 PMC4133404

[7] Chaikitgosiyakul S Rijken MJ Muehlenbachs A Lee SJ Chaisri U Viriyavejakul P A morphometric and histological study of placental malaria shows significant changes to villous architecture in both *Plasmodium falciparum* and *Plasmodium vivax* infection. Malar J (2014) 13:4. 10.1186/1475-2875-13-4 24386908 PMC3900675

[8] Datta M Biswas J Dasgupta S Banerjee K Choudhury S Sengupta SK Comparative study on antenatal and perinatal outcome of vivax and falciparum malaria in a Tertiary Care Hospital of Kolkata, India. J Clin Diagn Res (2017) 11:QC01–04. 10.7860/JCDR/2017/23051.9195 28274003 PMC5324448

[9] Miller LH Baruch DI Marsh K Doumbo OK. The pathogenic basis of malaria. Nature (2002) 415:673–9. 10.1038/415673a 11832955

[10] Rasti N Wahlgren M Chen Q. Molecular aspects of malaria pathogenesis. FEMS Immunol Med Microbiol (2004) 41:9–26. 10.1016/j.femsim.2004.01.010 15094163

[11] Korir CC Galinski MR. Proteomic studies of *Plasmodium knowlesi* SICA variant antigens demonstrate their relationship with *P. falciparum* EMP1. Infect Genet Evol (2006) 6:75–9. 10.1016/j.meegid.2005.01.003 16376842

[12] Mueller I Galinski MR Baird JK Carlton JM Kochar DK Alonso PL Key gaps in the knowledge of *Plasmodium vivax*, a neglected human malaria parasite. Lancet Infect Dis (2009) 9:555–66. 10.1016/S1473-3099(09)70177-X 19695492

[13] Maguire JD Baird JK. The ‘non-falciparum’ malarias: the roles of epidemiology, parasite biology, clinical syndromes, complications and diagnostic rigour in guiding therapeutic strategies. Ann Trop Med Parasitol (2010) 104:283–301. 10.1179/136485910X12743554760027 20659390

[14] Ebbinghaus P Krücken J Characterization and tissue-specific expression patterns of the *Plasmodium chabaudi cir* multigene family. Malar J (2011) 10:272 10.1186/1475-2875-10-272 21929749 PMC3189184

[15] Carvalho BO Lopes SC Nogueira PA Orlandi PP Bargieri DY Blanco YC On the cytoadhesion of *Plasmodium vivax*-infected erythrocytes. J Infect Dis (2010) 202:638–47. 10.1086/654815 20617923

[16] De las Salas B Segura C Pabón A Lopes SC Costa FT Blair S Adherence to human lung microvascular endothelial cells (HMVEC-L) of *Plasmodium vivax* isolates from Colombia. Malar J (2013) 12:347 10.1186/1475-2875-12-347 24080027 PMC3850517

[17] Chotivanich K Udomsangpetch R Suwanarusk R Pukrittayakamee S Wilairatana P Beeson JG *Plasmodium vivax* adherence to placental glycosaminoglycans. PLoS One (2012) 7:e34509. 10.1371/journal.pone.0034509 22529919 PMC3328474

[18] Marín-Menéndez A Bardají A Martínez-Espinosa FE Bôtto-Menezes C Lacerda MV Ortiz J Rosetting in *Plasmodium vivax*: a cytoadhesion phenotype associated with anaemia. PLoS Negl Trop Dis (2013) 7:e2155. 10.1371/journal.pntd.0002155 23593522 PMC3617122

[19] Lopes SC Albrecht L Carvalho BO Siqueira AM Thomson-Luque R Nogueira PA Paucity of *Plasmodium vivax* mature schizonts in peripheral blood is associated with their increased cytoadhesive potential. J Infect Dis (2014) 209:1403–7. 10.1093/infdis/jiu018 24415786

[20] Field JW Sandosham AA Fong YL The Microscopial Diagnosis of Human Malaria. Kuala Lumpur: Institute for Medical Research (1963).

[21] Baro B Deroost K Raiol T Brito M Almeida AC de Menezes-Neto A *Plasmodium vivax* gametocytes in the bone marrow of an acute malaria patient and changes in the erythroid miRNA profile. PLoS Negl Trop Dis (2017) 11:e0005365 10.1371/journal.pntd.0005365 28384192 PMC5383020

[22] Siqueira AM Magalhães BML Melo GC Ferrer M Castillo P Martin-Jaular L Spleen rupture in a case of untreated *Plasmodium vivax* infection. PLoS Negl Trop Dis (2012) 6:e1934 10.1371/journal.pntd.0001934 23272256 PMC3521714

[23] Lacerda MV Fragoso SC Alecrim MG Alexandre MA Magalhães BM Siqueira AM Postmortem characterization of patients with clinical diagnosis of *Plasmodium vivax* malaria: to what extent does this parasite kill? Clin Infect Dis (2012) 55:e67–74. 10.1093/cid/cis615 22772803

[24] del Portillo HA Fernandez-Becerra C Bowman S Oliver K Preuss M Sanchez CP A superfamily of variant genes encoded in the subtelomeric region of *Plasmodium vivax*. Nature (2001) 410:839–42. 10.1038/35071118 11298455

[25] Fernandez-Becerra C Pein O de Oliveira TR Yamamoto MM Cassola AC Rocha C Variant proteins of *Plasmodium vivax* are not clonally expressed in natural infections. Mol Microbiol (2005) 58:648–58. 10.1111/j.1365-2958.2005.04850.x 16238616

[26] Lopez FJ Bernabeu M Fernandez-Becerra C del Portillo HA. A new computational approach redefines the subtelomeric vir superfamily of *Plasmodium vivax*. BMC Genomics (2013) 14:8. 10.1186/1471-2164-14-8 23324551 PMC3566924

[27] Bernabeu M Lopez FJ Ferrer M Martin-Jaular L Razaname A Corradin G Functional analysis of *Plasmodium vivax* VIR proteins reveals different subcellular localizations and cytoadherence to the ICAM-1 endothelial receptor. Cell Microbiol (2012) 14:386–400. 10.1111/j.1462-5822.2011.01726.x 22103402

[28] Udomsangpetch R Thanikkul K Pukrittayakamee S White NJ. Rosette formation by *Plasmodium vivax*. Trans R Soc Trop Med Hyg (1995) 89:635–7. 10.1016/0035-9203(95)90422-0 8594679

[29] Chotivanich KT Pukrittayakamee S Simpson JA White NJ Udomsangpetch R. Characteristics of *Plasmodium vivax*-infected erythrocyte rosettes. Am J Trop Med Hyg (1998) 59:73–6. 10.4269/ajtmh.1998.59.73 9684631

[30] Lee WC Malleret B Lau YL Mauduit M Fong MY Cho JS Glycophorin C (CD236R) mediates vivax malaria parasite rosetting to normocytes. Blood (2014) 123:e100–9. 10.1182/blood-2013-12-541698 24652986 PMC4007619

[31] Zhang R Lee WC Lau YL Albrecht L Lopes SC Costa FT Rheopathologic consequence of *Plasmodium vivax* rosette formation. PLoS Negl Trop Dis (2016) 10:e0004912. 10.1371/journal.pntd.0004912 27509168 PMC4980013

[32] Op Den Kamp JAF Lipid asymmetry in membranes. Ann Rev Biochem (1979) 48:47–71. 10.1146/annurev.bi.48.070179.000403 382989

[33] Wu Y Tibrewal N Birge RB. Phosphatidylserine recognition by phagocytes: a view to a kill. Trends Cell Biol (2006) 16:189–97. 10.1016/j.tcb.2006.02.003 16529932

[34] Lhermusier T Chap H Payrastre B. Platelet membrane phospholipid asymmetry: from the characterization of a scramblase activity to the identification of an essential protein mutated in Scott syndrome. J Thromb Haemost (2011) 9:1883–91. 10.1111/j.1538-7836.2011.04478.x 21958383

[35] Elliott JI Surprenant A Marelli-Berg FM Cooper JC Cassady-Cain RL Wooding C Membrane phosphatidylserine distribution as a non-apoptotic signalling mechanism in lymphocytes. Nat Cell Biol (2005) 7:808–16. 10.1038/ncb1279 16025105

[36] Rysavy NM Shimoda LM Dixon AM Speck M Stokes AJ Turner H Beyond apoptosis: the mechanism and function of phosphatidylserine asymmetry in the membrane of activating mast cells. Bioarchitecture (2014) 4:127–37. 10.1080/19490992.2014.995516 25759911 PMC4914033

[37] Lang KS Lang PA Bauer C Duranton C Wieder T Huber SM Mechanisms of suicidal erythrocyte death. Cell Physiol Biochem (2005) 15:195–202. 10.1159/000086406 15956782

[38] Bratosin D Estaquier J Ameisen JC Montreuil J Molecular and cellular mechanisms of erythrocyte programmed cell death: impact on blood transfusion. Vox Sang (2002) 83:307–10. 10.1111/j.1423-0410.2002.tb05324.x 12617159

[39] Lang E Lang F. Triggers, inhibitors, mechanisms, and significance of eryptosis: the suicidal erythrocyte death. Biomed Res Int (2015) 2015:513518. 10.1155/2015/513518 25821808 PMC4364016

[40] Totino PR Daniel-Ribeiro CT Ferreira-da-Cruz MF. Evidencing the role of erythrocytic apoptosis in malarial anemia. Front Cell Infect Microbiol (2016) 6:176. 10.3389/fcimb.2016.00176 28018860 PMC5145864

[41] Totino PR Magalhães AD Silva LA Banic DM Daniel-Ribeiro CT Ferreira-da-Cruz MF. Apoptosis of non-parasitized red blood cells in malaria: a putative mechanism involved in the pathogenesis of anaemia. Malar J (2010) 9:350. 10.1186/1475-2875-9-350 21126362 PMC3017533

[42] Fendel R Brandts C Rudat A Kreidenweiss A Steur C Appelmann I Hemolysis is associated with low reticulocyte production index and predicts blood transfusion in severe malarial anemia. PLoS One (2010) 5:e10038. 10.1371/journal.pone.0010038 20386613 PMC2850371

[43] Wali RK Jaffe S Kumar D Kalra VK. Alterations in organization of phospholipids in erythrocytes as factor in adherence to endothelial cells in diabetes mellitus. Diabetes (1988) 37:104–11. 10.2337/diabetes.37.1.104 3335275

[44] Bonomini M Sirolli V Gizzi F Di Stante S Grilli A Felaco M. Enhanced adherence of human uremic erythrocytes to vascular endothelium: role of phosphatidylserine exposure. Kidney Int (2002) 62:1358–63. 10.1111/j.1523-1755.2002.kid560.x 12234306

[45] Setty BN Kulkarni S Stuart MJ. Role of erythrocyte phosphatidylserine in sickle red cell-endothelial adhesion. Blood (2002) 99:1564–71. 10.1182/blood.V99.5.1564 11861269

[46] Wautier MP Héron E Picot J Colin Y Hermine O Wautier JL. Red blood cell phosphatidylserine exposure is responsible for increased erythrocyte adhesion to endothelium in central retinal vein occlusion. J Thromb Haemost (2011) 9:1049–55. 10.1111/j.1538-7836.2011.04251.x 21362128

[47] Closse C Dachary-Prigent J Boisseau MR. Phosphatidylserine-related adhesion of human erythrocytes to vascular endothelium. Br J Haematol (1999) 107:300–2. 10.1046/j.1365-2141.1999.01718.x 10583215

[48] Manodori AB Barabino GA Lubin BH Kuypers FA. Adherence of phosphatidylserine-exposing erythrocytes to endothelial matrix thrombospondin. Blood (2000) 95:1293–300. 10666202

[49] Borst O Abed M Alesutan I Towhid ST Qadri SM Föller M Dynamic adhesion of eryptotic erythrocytes to endothelial cells via CXCL16/SR-PSOX. Am J Physiol Cell Physiol (2012) 302:C644–51. 10.1152/ajpcell.00340.2011 22173866

[50] Silverstein RL Baird M Lo SK Yesner LM. Sense and antisense cDNA transfection of CD36 (glycoprotein IV) in melanoma cells. Role of CD36 as a thrombospondin receptor. J Biol Chem (1992) 267:16607–12. 1379600

[51] Betal SG Setty BNY. Phosphatidylserine-positive erythrocytes bind to immobilized and soluble thrombospondin-1 via its heparin-binding domain. Transl Res (2008) 152:165–77. 10.1016/j.trsl.2008.07.007 18940719 PMC2628802

[52] Ohta K Fukuuchi Y Tomita M Tanahashi N Matsuoka S Takeda H. Monoclonal antibody against platelet thrombospondin decreases erythrocyte aggregation rate. Biorheology (1991) 28:551–6. 1818743 10.3233/bir-1991-28606

[53] Wun T Paglieroni T Field CL Welborn J Cheung A Walker NJ Platelet-erythrocyte adhesion in sickle cell disease. J Investig Med (1999) 47:121–7. 10198567

[54] Walker B Towhid ST Schmid E Hoffmann SM Abed M Münzer P Dynamic adhesion of eryptotic erythrocytes to immobilized platelets via platelet phosphatidylserine receptors. Am J Physiol Cell Physiol (2014) 306:C291–7. 10.1152/ajpcell.00318.2013 24284794

[55] Joshi P Dutta GP Gupta CM. An intracellular simian malarial parasite (*Plasmodium knowlesi*) induces stage-dependent alterations in membrane phospholipid organization of its host erythrocyte. Biochem J (1987) 246:103–8. 10.1042/bj2460103 3675550 PMC1148245

[56] Schwartz RS Olson JA Raventos-Suarez C Yee M Heath RH Lubin B Altered plasma membrane phospholipid organization in *Plasmodium falciparum*-infected human erythrocytes. Blood (1987) 69:401–7. 3542079

[57] Lang F Lang PA Lang KS Brand V Tanneur V Duranton C Channel-induced apoptosis of infected host cells – the case of malaria. Pflugers Arch (2004) 448:319–24. 10.1007/s00424-004-1254-9 15042371

[58] Butthep P Wanram S Pattanapanyasat K Vattanaviboon P Fucharoen S Wilairat P. Cytoadherence between endothelial cells and *P. falciparum* infected and noninfected normal and thalassemic red blood cells. Cytometry B Clin Cytom (2006) 70:432–42. 10.1002/cyto.b.20141 16977636

[59] Alesutan I Bobbala D Qadri SM Estremera A Föller M Lang F Beneficial effect of aurothiomalate on murine malaria. Malar J (2010) 9:118 10.1186/1475-2875-9-118 20459650 PMC2875225

[60] Eda S Sherman IW. Cytoadherence of malaria-infected red blood cells involves exposure of phosphatidylserine. Cell Physiol Biochem (2002) 12:373–84. 10.1159/000067908 12438774

[61] Balaji SN Trivedi V Extracellular methemoglobin primes red blood cell aggregation in malaria: an *in vitro* mechanistic study. FEBS Lett (2013) 587:350–7. 10.1016/j.febslet.2012.12.015 23313944

[62] Franke-Fayard B Janse CJ Cunha-Rodrigues M Ramesar J Büscher P Que I Murine malaria parasite sequestration: CD36 is the major receptor, but cerebral pathology is unlinked to sequestration. Proc Natl Acad Sci U S A (2005) 102:11468–73. 10.1073/pnas.0503386102 16051702 PMC1183563

[63] Lovegrove FE Gharib SA Peña-Castillo L Patel SN Ruzinski JT Hughes TR Parasite burden and CD36-mediated sequestration are determinants of acute lung injury in an experimental malaria model. PLoS Pathog (2008) 4:e1000068. 10.1371/journal.ppat.1000068 18483551 PMC2364663

[64] Serghides L Smith TG Patel SN Kain KC CD36 and malaria: friends or foes? Trends Parasitol (2003) 19:461–9. 10.1016/j.pt.2003.08.006 14519584

[65] van Schravendijk MR Handunnetti SM Barnwell JW Howard RJ. Normal human erythrocytes express CD36, an adhesion molecule of monocytes, platelets, and endothelial cells. Blood (1992) 80:2105–14. 1382721

[66] Arman M Adams Y Lindergard G Rowe JA. A method for positive and negative selection of *Plasmodium falciparum* platelet-mediated clumping parasites and investigation of the role of CD36. PLoS One (2013) 8:e55453. 10.1371/journal.pone.0055453 23405153 PMC3566186

[67] Mota MM Jarra W Hirst E Patnaik PK Holder AA *Plasmodium chabaudi*-infected erythrocytes adhere to CD36 and bind to microvascular endothelial cells in an organ-specific way. Infect Immun (2000) 68:4135–44. 10.1128/IAI.68.7.4135-4144.2000 10858230 PMC101711

[68] Frech C Chen N. Variant surface antigens of malaria parasites: functional and evolutionary insights from comparative gene family classification and analysis. BMC Genomics (2013) 14:427. 10.1186/1471-2164-14-427 23805789 PMC3747859

[69] Ruggeri ZM Von Willebrand factor, platelets and endothelial cell interactions. J Thromb Haemost (2003) 1:1335–42. 10.1046/j.1538-7836.2003.00260.x 12871266

